# DNA sequence-dependent chromatin architecture and nuclear hubs formation

**DOI:** 10.1038/s41598-019-51036-9

**Published:** 2019-10-10

**Authors:** Kamel Jabbari, Maharshi Chakraborty, Thomas Wiehe

**Affiliations:** Institute for Genetics, Biocenter Cologne, University of Cologne, Zülpicher Straße 47a, 50674 Köln, Germany

**Keywords:** Genetics, Computational biology and bioinformatics, Genetics, Computational biology and bioinformatics

## Abstract

In this study, by exploring chromatin conformation capture data, we show that the nuclear segregation of Topologically Associated Domains (TADs) is contributed by DNA sequence composition. GC-peaks and valleys of TADs strongly influence interchromosomal interactions and chromatin 3D structure. To gain insight on the compositional and functional constraints associated with chromatin interactions and TADs formation, we analysed intra-TAD and intra-loop GC variations. This led to the identification of clear GC-gradients, along which, the density of genes, super-enhancers, transcriptional activity, and CTCF binding sites occupancy co-vary non-randomly. Further, the analysis of DNA base composition of nucleolar aggregates and nuclear speckles showed strong sequence-dependant effects. We conjecture that dynamic DNA binding affinity and flexibility underlay the emergence of chromatin condensates, their growth is likely promoted in mechanically soft regions (GC-rich) of the lowest chromatin and nucleosome densities. As a practical perspective, the strong linear association between sequence composition and interchromosomal contacts can help define consensus chromatin interactions, which in turn may be used to study alternative states of chromatin architecture.

## Introduction

Recently developed chromatin conformation capture techniques and methods uncovered principles of the spatial organization of nuclear hubs and interchromosomal interactions. The discovery, characterization, and function of chromatin domains have been covered by a number of reviews^1–5^. These methods revealed many features of 3D genome organization, in particular, topologically associated domains (TADs)^6,7^, self-interacting regions, characterized by frequent within-chromatin interactions compared to relatively lower-frequency interactions with surrounding regions. They represent genomic architectural modules that constrain enhancer-promoter contacts, thereby setting tissue-specific interactions that regulate gene expression within TADs and connecting chromatin architecture with local gene expression^2,8^. TADs may contain smaller “sub-TADs”^9,10^ and, at smaller scale, may harbour individual “loops”^9^ or “insulation neighbourhoods”^8,11^.

The initial definition of TADs included implicitly Lamina Associated Domains (LADs)^12^. LADs occupy ~40% of the chromatin space, they are known to be AT-rich^13^ and recent work showed that mammalian interphase chromatin is a mosaic of different TADs (or inter-LADs) and LADs, broadly mapping to GC-rich and GC-poor chromosomal domains (isochores); constitutive LADs (cLADs) being the GC-poorest^14^. These investigations led to the following observations: (1) the match between isochores and TADs; (2) the evidence of complex structure and high CTCF binding sites of GC-rich TADs, compared to rather flat GC profiles of LADs; (3) the correspondence between GC valleys/peaks and chromatin loops (see Fig. 3 in^14^) (4) the qualitative assessment of preferential interchromosomal interactions among GC-rich TADs, where chromatin is open and negative super-coiling is more frequent^15,16^. Yet convincing quantitative evidence on the role of sequence composition in interchromosomal interactions is still scarce.

Within the cell nucleus, contacts between genomic regions associated with the nuclear lamina occur between domains that are intra-chromosomally close to each other^17,18^, with preferences for interactions among GC-poor regions for larger intra-chromosomal distance^14^. GC-rich, gene-rich regions show greater compositional heterogeneity and overall weaker intra-chromosomal interactions than loci in GC-poor, gene-poor regions. The intensity of such interactions however exhibits significant change from cell to cell, *e*.*g*., between growing and senescent cells^19^.

Lately, Quinodoz *et al*.^20^ developed a method called split-pool recognition of interactions by tag extension (SPRITE), which led to the discovery of two major hubs of interchromosomal interactions arranged around nuclear bodies: the nucleolus and the nuclear speckles. The authors concluded that inactive hub regions are much closer to the nucleolus and that 3D distance of DNA regions to these hubs is based on their functional properties, including the density of active Pol II within interacting genomic regions. Moreover, a large fraction of genomic regions showed preferential contacts with either hub; chromosomal regions that frequently contact the nucleolar hub were under-represented relative to the nuclear speckle hub, and *vice versa* (anti-correlated). Here we demonstrate that this anti-correlation is strongly associated with DNA sequence composition of the loci under consideration. We suggest that regional GC-peaks and valleys, together with the flat GC profile of LADs, contribute to the encoding of higher order interchromosomal hubs. To further explain the dependence of higher chromatin organization on sequence composition, we studied the compositional gradients within TADs and quantified the intra-TADs sequence composition and its co-variation with the density of genes, of CTCF binding sites and of super-enhancers (SE); the latter are able to drive higher levels of transcription than single/typical enhancers^21,22^. To better understand the role of transcription activity in nuclear molecular crowding, we estimated inter- and intra-TADs gene transcriptional profiles using 27 human tissues. Together, this analysis suggests that physicochemical and functional constraints affect chromatin loops formation and may induce phase separation through loop clusters interconnections. In such a model, multivalent macromolecular interactions^23^ are favourably occurring in GC-rich, nucleosome free chromatin^24–26^.

## Results

### Interchromosomal interactions are sequence composition dependent

**Figure 1:**
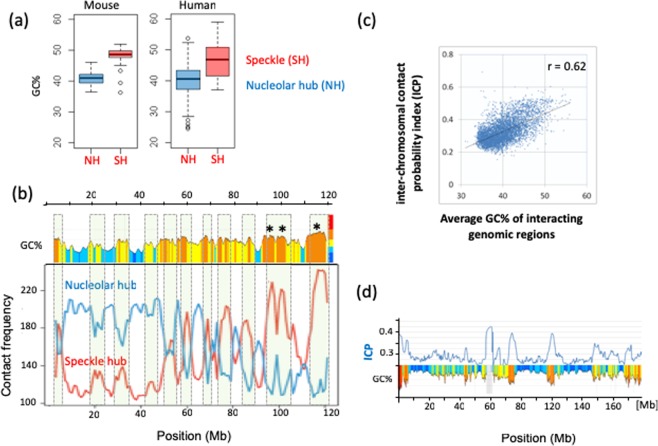
Contact frequencies of nucleolar and speckle hubs are GC dependent. (**a**) Boxplots of GC% in regions of annotated genomic DNA defined as inactive/nucleolar hubs (in blue) or active/speckle hubs (in red) in mouse ES cells and human GM12878 cells at 1 Mb resolution. Differences in GC% between the potential hub regions in human and mouse were tested (*p*-values < 2.2e^−16^ using Student’s *t-test*). (**b**) Compositional profile and SPRITE identified interactions across mouse chromosome 11 (blue and red lines are taken from Fig. 6b in Quinodoz *et al*.). Interchromosomal interactions associated with inactive hub (blue) and with active hub (red). The multi-coloured compositional profile represents increasing GC% (see colour code bar right to the GC profile) in the order, deep blue (33–37%GC), light blue (37–41%GC), yellow (41–46%GC), orange (46–53%GC) and red (53–59%GC). Grey bars highlight anti-correlations. Asterisks refer to the observed speckle hub regions as indicated by Quinodoz *et al*. (**c**) Human genome wide correlation of contact probability index (ICP) and regional GC%. (**d**) Sliding window profiles of GC% and ICP across human chromosome 7. The grey bar centred at 60 Mb corresponds to the centromere sequencing gap.

To study the effect of regional genomic GC level on interchromosomal interactions, including hub formation, we first used the data provided by Quinodoz *et al*. to show that there is a strong GC enrichment of interchromosomal hubs arranged around the nuclear speckles (Fig. 1a). These associations (Pearson r = 0.82, *p*-values < 2.2e^−16^) are also observed locally along non-contiguous regions of mouse chromosome 11 reported in Quinodoz *et al*. (Fig. 1b). These results suggest that the preferential spatial arrangement of either the nucleolus or nuclear speckle hubs can be recognized by GC level changes along chromosomes.

Using a different method to explore interchromosomal interactions, Kalhor *et al*.^27^ devised tethered chromosome conformation capture (TCC) method and showed that specific clusters of functionally active loci are more likely to form interchromosomal contacts than inactive ones and that most of these contacts are a result of encounters between loci that are accessible to each other and have higher RNA polymerase II binding. Importantly, and in agreement with the SPRITE results, our analysis of genome-wide TCC data shows that interchromosomal interaction probability (ICP) is highly correlated (Pearson r = 0.62, *p*-value < 2.2e^−16^) with GC% of the interacting domains (Fig. 1c). This strong correlation is obvious when both ICP and GC% are visualized as profiles along chromosomes, as shown for human chromosome 7 (Fig. 1d); demonstrating clearly that interchromosomal contacts among GC-rich TADs are substantially high. This indicates that fitting a linear equation/regression to observed data is a good model for estimating either the expected interaction intensity or the expected GC content of the interacting DNA segments. Last, one can also notice the high interaction of centromeric regions, increased ICP values may be due to repetitive “satellite” DNA embedding centromeres and the frequent inter-centromeres clusters, thought to initiate the formation of nucleoli and nuclear radial position^28^.

The presence of GC-peaks and GC-valleys (Fig. 1b,d) along chromosomes appears to be a characteristic property of interchromosomal interactions. This prompted us to have a closer look at individual patterns of GC change within TADs or loops, and to quantify the intra-TAD distributions of key functional elements associated to these variations, namely genes, CTCF binding and super-enhancers densities across TADs.

### Functional features of TADs and loops

**Figure 2:**
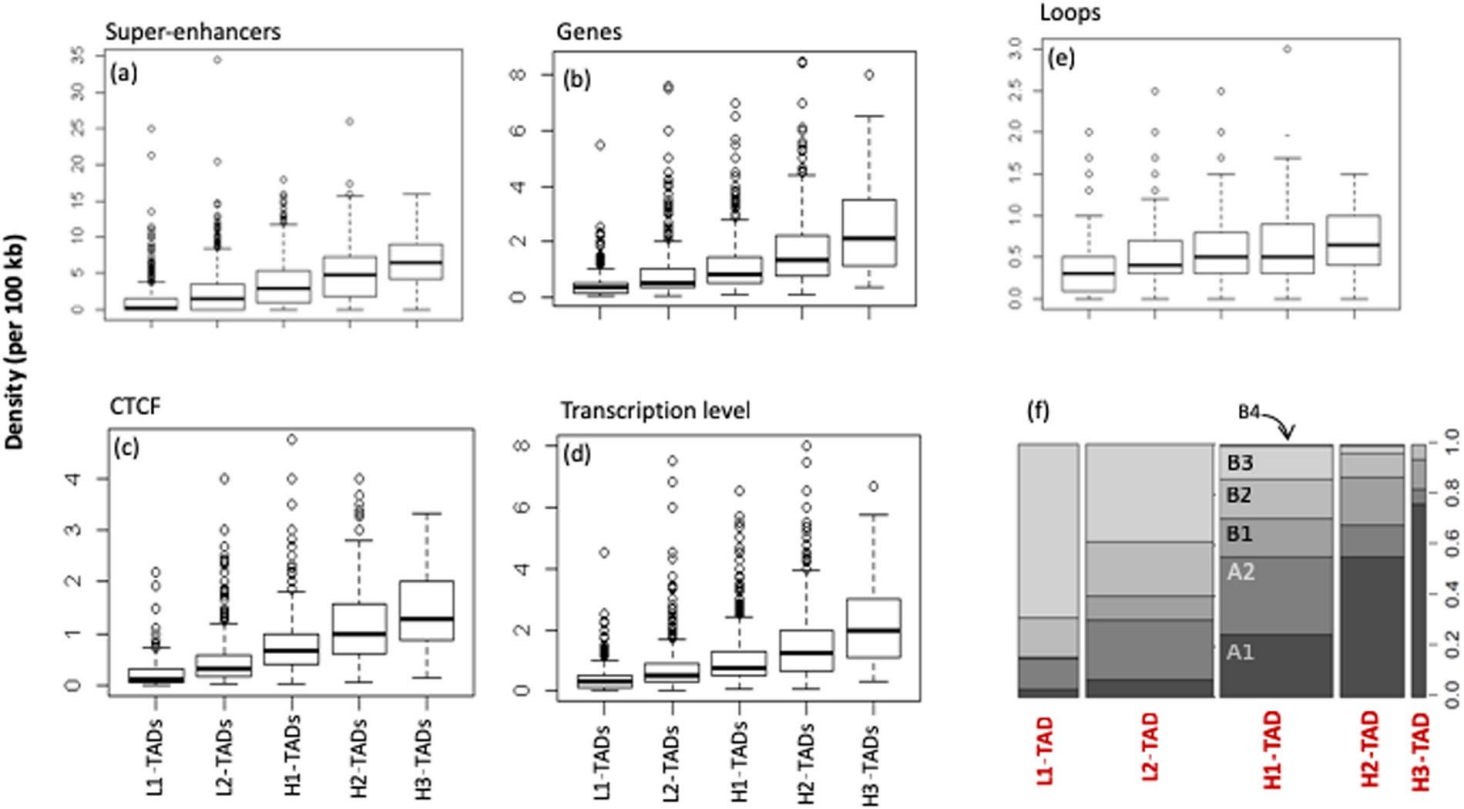
Increase of loops, genes densities, transcription level, super-enhancers and CTCF binding frequencies per 100 kb, from L1-TADs (GC-poor) to H3-TADs (GC-richest). GC-rich TADs are more gene dense and transcriptionally more active than GC-poor TADs. Chromatin sub-compartments A1 and A2 are enriched in H2/H3-TADs (f); the different sizes of the rectangles in (f) reflect the proportion of TADs families in the human genome.

GC-rich TADs exhibit higher frequency of loops or sub-TADs (Fig. 2e). The increase of GC level of TADs is also accompanied by increased gene density, CTCF binding, SE ovelap frequencies and transcription level (Fig. 2a–d). Open chromatin sub-compartments^10^ A1 and A2 are enriched in H2/H3-TADs (Fig. 2f), more compact B1-B3 sub-compartments are biased towards L1/L2-TADs (mainly LADs); B4 sub-compartment is a chromatin state that is specific to chr19, a chromosome composed mainly of GC-rich, gene-rich isochores and almost with no anchor to the lamina.

### Intra-TADs compositional shape and chromatin loops heterogeneity

Having observed a very strong correlation between physical contacts and DNA sequence composition (GC%), we wanted to know how does sequence composition vary within TADs. Based on the GC profiles of size binned TADs and loops, and ignoring orientation (equivalence of 5′ or 3′ gradient patterns), we defined six possible classes of loops or TADs; A (increasing or decreasing GC%), B (bell shape or peak), C (valley shape), B^−^ (half bell), C^−^ (half valley) and D (uncorrelated). (for details see Methods section) These were found to be non-uniformly distributed across the human genome (Fig. S2). B class density is highest in GC-rich TADs and represents 45% of all classes in H2-H3 TADs, whereas C classes are most frequent in GC-poor TADs and represent 32% of all classes in L1-TADs. B^−^ and C^−^ are quasi-B and quasi-C TADs (See also legend to Fig. S1), their genome wide frequency distributions across the TADs GC range follow the same pattern as B and C TADs. D class (flat or spiky GC profile, −0.4 < r < 0.4) is homogenously (~25%) distributed (Fig. S2a). Notice that D class GC variation along L1 and L2-TADs are more homogenous than those from H2-H3, as one would expect from the positive correlation between average GC% and its standard deviation^29^. To see wether TADs/loops form clusters based on their internal variation of GC%, we performed unsupervised Principal Component Analysis (PCA) on the raw matrices having rows as binned GC values across individual TADs/loops and columns as binned TADs/loops length (see Methods for more details). Expectedly, PCA results showed that F1 values are highly correlated (r = 0.99) with the average GC% of TADs/loops. We thus used F2 and F3 principal components and could clearly identify separate clusters, B (bell shape) and B^−^ (half bell shape) classes, on one hand and C (valley shape) and C^−^ (half valley shape) classes on the other (Fig. S2b). This indicates that indeed, the variance of GC within loop domains (intra-loop) explains the positioning of individual loops in the (F2, F3) PCA plane (see Table S1 for genome wide proportions of all TADs and loops classes). In view of the nested structure of TADs, one of the sources of uncertainties associated TADs boundaries^30^, we re-estimated the relative contribution of each class of the TADs/loops after either increasing or decreasing their size by 50 kb on both 3′ and 5′ ends, then their genome wide frequencies were recalculated (Table S2). Interestingly, a majority of loops remained in C, B^−^, C^−^ classes, these are in other words the least sensitive to boundaries definition, whereas a fraction of B-loops moved to the B^−^ class. This again indicates that classes B^−^ and C^−^ behave like classes B and C, respectively; they together represent ~70% of the annotated TADs (see Table S1). In what follows, we will focus B and C classes, due to their sharp separation in the PCA and their shared GC-gradients with B^−^ and C^−^.

### Intra-TADs functional features

**Figure 3:**
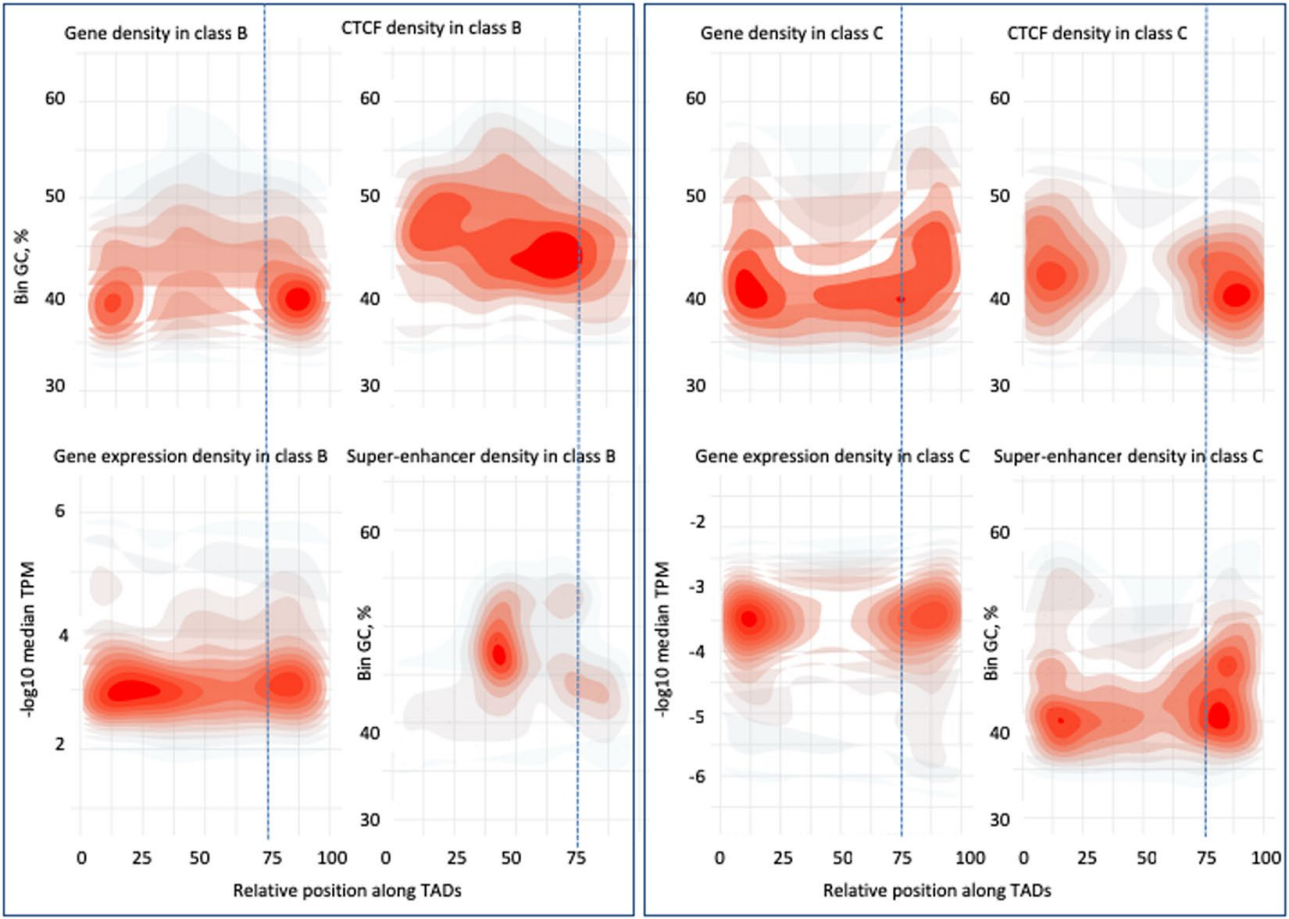
Kernel density plots showing distributions of genes, log_10_ [mean transcripts per million (TPM)], super-enhancers and CTCF binding sites within class B and class C TADs. Red contours indicate high density of points whereas grey contours indicate lower density. The border of C-TADs are gene, CTCF binding and transcription dense. Only SE overlap frequency is GC dependent in the case of B-TADs. Dotted lines mark the 75% bin, it points to the shift in density at the TADs border, in particular, the shift in CTCF density between B and C-TADs.

B and C-TADs (Fig. 3) exhibit different patterns of functional elements distributions; genes, CTCF binding and SE overlap are high at the GC-rich borders of the C-TADs. B-TADs show a less expected pattern, the gene density is highest at borders despite their relative GC-poorness compared to the TAD centres, and CTCF binding density is diffuse instead of peaking at the centre of the B-TADs, where GC% is the highest.

Expression data across 27 tissues showed that GC-rich TADs are enriched for highly expressed and housekeeping genes (Fig. S3), making them poised to contribute more to active hub regions, such as nuclear speckles (Fig. 1a,b). B^−^ and C^−^ show similar density patterns to those of B and C TADs and A-TADs and D-TADs behave similarly by exhibiting gene dense borders (Fig. S4). Enhancers frequency almost invariably follow the GC-gradient of TADs, in agreement with the overall (inter-TAD) trend observed in Fig. 2. Notably, C TADs exhibit a lower log_10_ [mean TPM] value compared to B TADs.

So far, the GC level of TADs was shown to correlate positively with other functional features, such as gene expression, gene density, SE overlap frequency and CTCF site occupancy. CTCF and SMC cohesin complex are associated with insulator function and are found at TAD boundaries^6,7^. Such an organization is most evident for C TADs, along which the frequency of CTCF binding, of genes and of SE are peaking at the chromatin domains borders. The characteristic shift of high density of CTCF binding sites in B-TADs (Fig. 2), fits with its GC-rich centre (CTCF binding sites are themselves GC-rich) and may in turn explain the propensity of B-TADs to harbour multiple loops, either nested or neighbouring each other, as suggested by higher loop density in GC-rich TADs (Fig. 2e). Incidentally, when we analysed mouse liver cells for which TADs and sub-TADs data were available^31^, compared to C-TADs, B-TADs showed a significant enrichment for these substructures (3.66 times enrichment, t-test *p*-value = 0.001), similar overrepresentation (3.59 times, t-test *p*-value = 0.001) of sub-TADs is also observed for B^−^ TADs. The sub-TAD structure appears therefore, in part favoured by the local high density of CTCF binding sites within B-TADs.

**Figure 4:**
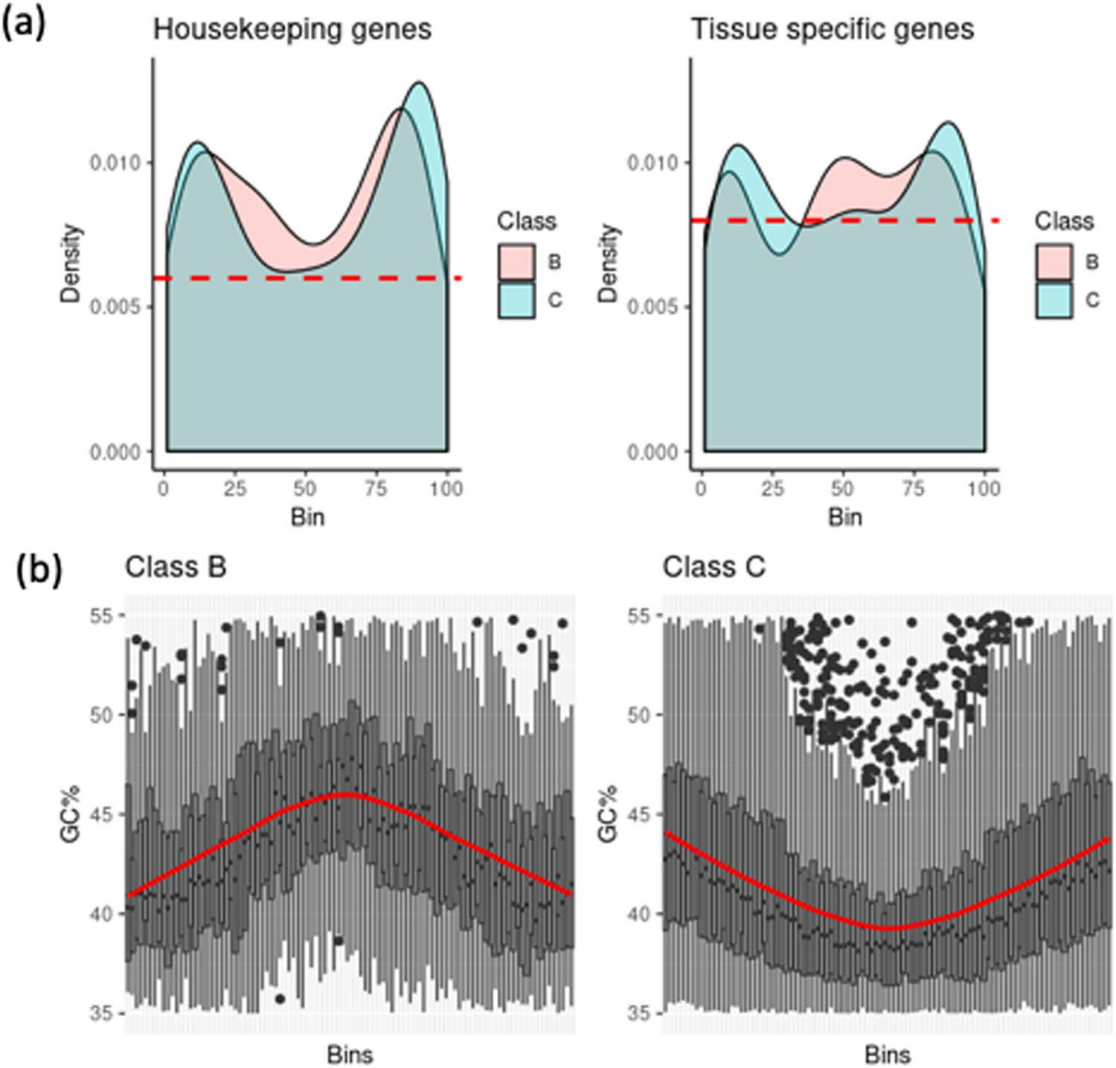
Tanscriptional activilty across TADs/loops. (**a**) Frequency density plots of housekeeping genes and tissue-specific genes across B and C-TADs using Tau metric. (**b**) GC% across B and C-TAD classes. Both housekeeping and tissue-specific gene densities are high at TADs borders. The density of tissue specific genes is high in the middle of class B.

B-TADs harbour relatively more housekeeping genes than C-TADs (Wilcoxon rank test, *p*-value = 0.036); the same trend can be observed for class B^−^ compared to class C^−^, although with a Wilcoxon rank test *p*-value of 0.097 (Fig. S5). Interestingly, both housekeeping and tissue-specific gene densities are highest at TAD-borders, but the density of tissue specific genes can also be high in the middle of class B-TADs (Fig. 4). This result is in part in agreement with the observation that boundary regions are enriched for housekeeping genes^6^.

## Discussion

### Constrained interchromosomal interactions

Up to this point, we could show (1) a strong compositional anti-correlation between active and inactive hub regions, and the increased bias of contact probability index values towards GC-rich TADs or isochores (Fig. 1); (2) the existence of TADs/loops classes, supported by supervised and unsupervised analysis of their compositional profiles; (3) that the density distributions of these classes across the genome (Fig. S2) are non-uniform. C/C^−^ class is biased towards GC-poor TADs, the latter will consequently tend to form nucleolar hubs, or interact at the nuclear envelope, to which AT-rich regions are regularly tethered. It is not clear if these silenced chromatin clusters are actively self-maintained or if the cell expression program primarily sets favourable nucleation around active hubs; in line with the second possibility, Pol II transcripts derived from intronic *Alu* elements (which are transcribed in the GC-rich nuclear interior) accumulate in nucleoli and were reported to be important for nucleolar integrity^32^. The inactive compartment of cLADs (GC-poorest TADs) is maintained by preferential sequestration in the nuclear envelope neighbourhood, while GC-rich TADs, transcriptionally active and mechanically flexible/softer^33^, may need active self-maintenance. Transcriptionally active TADs correspond to A1 + A2 open chromatin sub-compartments (Fig. 2f) and are generally located in the nuclear interior^34,35^, consistent with a less compact organization and an enrichment of long-range chromosomal contacts with other active TADs and potentially multi-TAD hubs^36,37^.

From a physicochemical point of view, interchromosomal interactions may recruit TADs and sub-TADs or loops with different compositional patterns (*e*.*g*. peak and valley) and consequently with different propensities to form nucleosomes and distinct abilities to bend and curve. In fact, GC content and dinucleotides frequencies may impose DNA structural/conformational constraints^38,39^; AT-rich tracts, AA/TT dinucleotide and AAA/TTT trinucleotide frequencies can rise the stiffness of the DNA fibre^40^ and GC tracts as well as the frequency of AAAA tetranucleotides can explain more that 50% of the variation in nucleosome occupancy^41,42^. Next to these DNA sequence factors, other histone marks are surrogates for transcriptional activity that can impact local chromatin structure.

**Figure 5:**
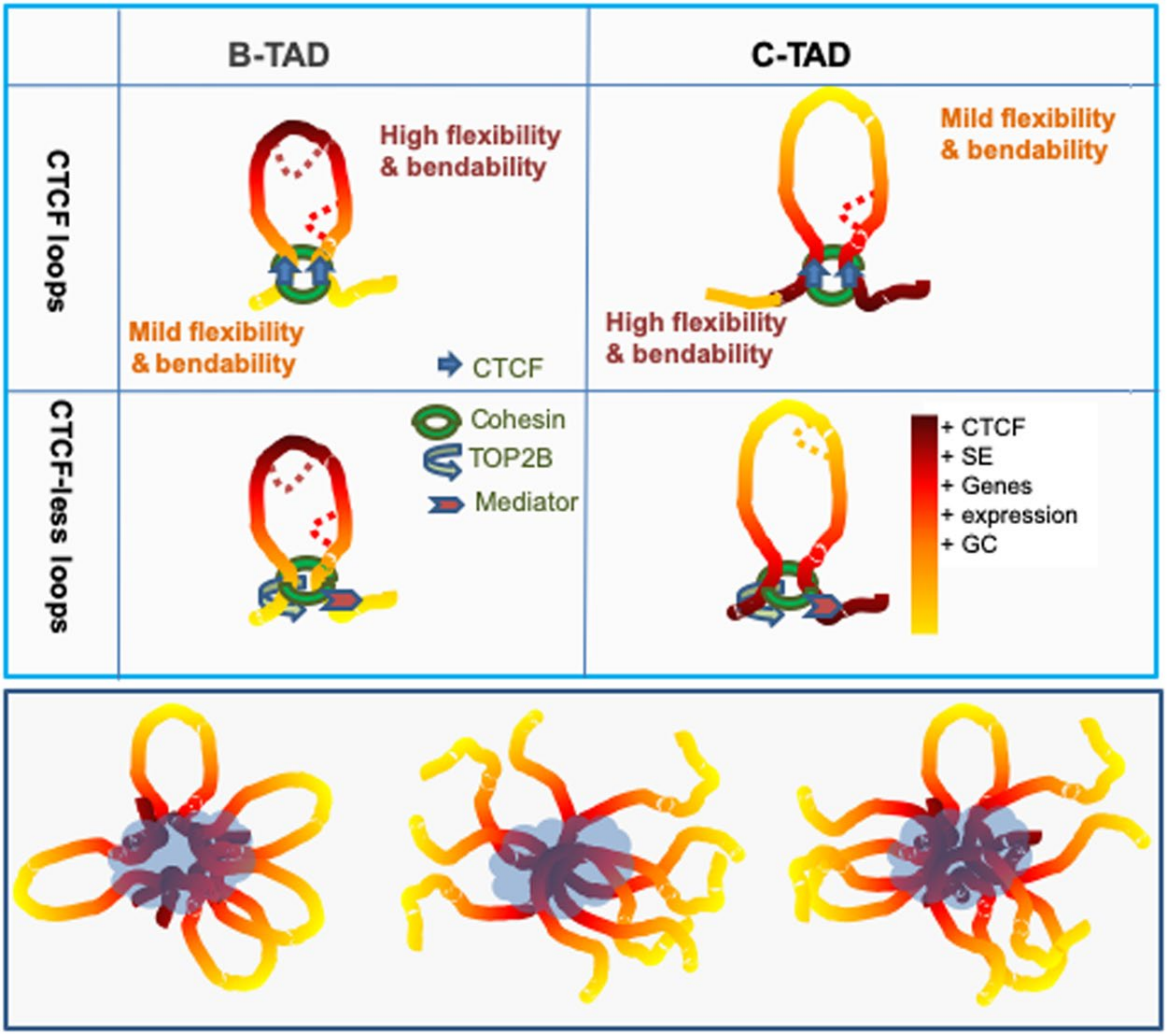
Cartoon depicting idealised chromatin fibre accounting for the formation TADs and chromatin hubs. (Top panel) Chromatin fibre with GC-peak and GC valley; the yellow to dark red colour gradient refers to increased frequency of GC-kmers. Dotted line corresponds to sub-TADs or sub-loops. Depending whether the TAD belongs to B or C-class, the initiation step of loop formation may follow from local flexibility/stiffness of the DNA fibre (see discussion) and nucleosome density dependent events. Because the stiffer the DNA is, more difficult it is to form small TADs/loops, B-TADs are drawn smaller than C-TADs. The formation of CTCF-less TADs, may be mediated by cohesin (green ring), mediator complex (in pink), or other multiple co-activators and general transcription factors. Top2b (in blue) is known to co-bind DNA sites with cohesin in CTCF-less loops. (Bottom panel) Petal (C-loops), reverse-petal like (B-loops) and mixed arrangement of GC-rich loop condensates in mechanically soft, low-density genomic regions associated with active gene expression. Compositional constraints are expected to counter non-specific interactions and leads to pulling out relatively GC-poor regions of the genome (heterochromatin-rich). In addition to agreeing with the hypothesis of Shin *et al*. and Hniz *et al*., our model stresses the link between compositional constraints and the associated stiffness landscape, on one hand, and the ensuing cooperative mechanism of loops interactions, on the other. Intrinsically protein disordered domains and a variety of TFs and histone modifications are contributing to the process of phase separation (blue cloud). Note that this is a simplified representation, it shows only B and C TAD types combinations, quantitative combination of other TAD classes are also possible.

More relevant to the large-scale GC variations, electro-kinetic DNA stretching^43^ showed that a quantifier of the stiffness of polymers, the persistence length of long DNA (>100 kb) has a remarkable dependence on the underlying sequence; rigid and unbent structures are AT-rich as opposed to GC-rich ones^43^. These differences and their possible consequences on genome folding are pictured in Fig. 5.

In the case of C-TADs endowed with CTCF, the crest mild flexibility and within C-TAD compositional design can be accommodated by single cohesin ring sliding over a preformed loop, through a reeling/extrusion mechanism^44–48^ or a handcuff cohesin rings, which according to the “handcuff model”^49^, could entrap single chromatin fibre (~10 nm diameter) connected at the loop base by a mediator. The high ATP cost associated with loop formation by extrusion^50–52^ is to be contrasted with recent observations that cohesion translocation occurs via diffusion, which does not require ATP^52,53^; hence, if any, this energetic burden is expected to be strongly reduced in CTCF-less loops.

The results presented here led to a model for the formation of LADs and TADs. LADs tend to be in a relatively unconstrained chromatin configuration, with an elongated shape and reduced flexibility compared to GC-rich gene-rich TADs^54,55^. The increase of AT-rich oligomers may reduce local DNA flexibility and bending of LADs, such sequence motifs may serve as points of nucleation for lamina and membrane proteins in less active chromatin domains, which generally harbour tissue-specific genes (Fig. S5). Depending whether a TAD or loop belongs to B or C-class, the initiation step of their formation may follow from local stiffness/bending of the DNA fibre, and nucleosome density, as early proposed for meiotic loops^55,56^. In line with this rationale, it was proposed^57^ that within TADs, nucleosome spacing, and DNA flexibility are higher in the middle than at the boundary of TADs. According to these authors, “attractive” forces within the chromatin domains can then confer specific local interactions, yielding a joint “insulation-attraction”. In contrast, this model did not consider sequence composition as a possible underlying cause^14^. In this regard, an explanation was put forward^58^, according to which TADs bending results from increasing GC% (and its correlates, oligo-Gs, CpGs and CpG islands, nucleosome spacing) that tips at the centre of loop (bell shape), this “moulding step” is followed by an “extruding step” that ends at the CTCF binding sites located at the base of the loops. This explanation is possible for a fraction (~10%, Table S1) of B-loops, namely those with GC% peaking at the centre and CTCF insulation at the base, but it does not apply to other TAD or loop classes. For instance, C class loops are five times more frequent than B class loops, their GC gradient is peaking at the borders, favouring CTCF binding and higher gene density (Fig. 3). In fact, independently of the GC gradients profiles, TADs/loops are able to self-interact and may form through the process of loop extrusion in the case of CTCF-cohesin loops, but CTCF-less loops need other mechanisms to account for their formation. Indeed, up to 62% of total identified CTCF-cohesin complexes are not associated with the anchor regions of a Hi-C loops, and 32% of TADs can form without an accompanying complex^59^. CTCF depletion^60–63^ reduced intra-TAD interactions and increased inter-TAD interactions, suggesting weakening, but not vanishing of TAD boundaries. In the absence of CTCF insulating factor, the presence of cohesin, mediator, or general transcription factors, may suffice for moderate chromatin folds insulation. Interestingly, CTCF-less loops consistently showed lower insulation of chromatin contacts^31^ and higher cohesin and TOP2B binding sites; TOP2B may facilitate supercoiling in a transcription-dependent manner^64,65^. This is in agreement with the observation that TOP2-mediated DNA fragility is linked to transcription and proximity to loop anchors^66^.

The expected lower bendability of C-TADs may underlie the fact that they are less dense in loops or sub-TADs compared to B-TADs. L1-TADs are expectedly more GC-homogenous than H2 + H3 TADs (Fig. S6), making the later more subject to local (within TAD/loop) bending and variable nucleosome density^24^ and supercoiling^15^.

### A compositional phase separation model of chromatin hubs

It is known that sub-cellular liquid-like compartments are selectively permeable to macromolecules and can regulate biochemical reactions by concentrating enzymes and substrates^67^. As far as transcriptional activity is concerned, Shin *et al*. 2018 proposed that growing nuclear condensates/hubs tend to physically exclude chromatin leading to droplets formation. Along this line, we argue that GC-rich loops will favour transcriptional condensates (Fig. 5, bottom panel), possibly through nanoscale transcriptional assemblies at enhancer-rich and multi-gene clusters^68,69^.

The anticorrelated compositional profiles described for active and inactive hubs (Fig. 1) and the intra-TAD variations in enhancers density and gene transcription, are reminiscent of the existence of meta-stable chromatin interactions that involve cooperative interaction between enhancer components and DNA base composition. According to this hypothesis, active molecular assemblies over the nuclear space are biased towards GC-rich TADs and loops. GC-poor TADs, which include constitutive and facultative LADs, are likely to communicate at the vicinity of the nuclear envelope or to cluster in nucleolar bodies (Fig. 1). Indeed, LADs display a substantial overlap with nucleolus-associated chromatin domains^70^. These observations appear to fit a “compositional phase-separation” model where multivalency, *i*.*e*. the availability of many different binding sites on a polymer^71^, is crucial. In the case of double stranded DNA (dsDNA); base stacking interactions^72,73^,key elements of DNA structure, are sequence dependent and determine the DNA flexibility and its phase behaviour^74^. This phenomenon may be related to sequence-dependent persistence length and bendability of GC-tracts^43^, which, at a critical threshold may lead to secondary phase separation, giving rise to liquid-crystalline dsDNA sub-compartments within droplets^75^. Accordingly, small droplets can nucleate in both low (GC-rich) and high chromatin density regions (GC-poor) and their growth will be enhanced in mechanically soft regions (GC-rich) of the lowest chromatin and nucleosome densities^33,76^, hence pulling distal GC-rich regions of the genome into confined nuclear space, while excluding background chromatin^33,77^.The present model does not exclude mechanisms for local hubs formation other than phase separation, the compositional heterogeneity of mammalian genomes and the associated differential nucleosome density may suffice to trigger molecular crowding, in particular within CG-rich chromatin domains, likely recruiting denser transcription factorties^78^, due to their high gene density and transcriptional activity.

Finally, considering the compositional profiles of TADs, B and C classes do not only differ in their GC-gradients; B-TAD centres exhibit high overlap with SEs, while C-TADs exhibit high SE overlap at the borders (Fig. 3). If SE concentration and gene expression clusters contribute to the valency of interacting chromatin segments, increasing the number of the SE in GC-rich TADs (Fig. 5, bottom panel), will promote the formation of increasingly larger complexes that will emerge as phase separated macromolecular entities such as speckles. Of note, intrinsically disordered domains from Mediator, Brd4, Oct4 or other TFs, are expectedly contributing to this process^68,69,77^.

## Conclusions

In summary, our results indicate that sequence composition is a key aspect of chromatin TADs and hub formations. Other large-scale correlates, such as gene density and protein-DNA binding affinities, also contribute to spatial organization and local concentrations around nuclear bodies. The initiation step for TAD or loop formation is under “compositional constraints”, essentially driven by local flexibility or stiffness of the coiled DNA fibre. In such a context, intrinsic properties of DNA sequence, bendability, and binding affinity of promoters and enhancers, may have a strong influence on TAD dynamics and the phase separation behaviour of chromatin. The formation of active chromatin assemblies is compositionally biased and may take place in both GC-rich and GC-poor chromosomal environments, but gains strength in mechanically soft regions (GC-rich), where DNA-protein foci coalesce via multivalent links. Interactions among and within chromatin domains can be viewed as part of a flexible “chromatin code”^79^ that can help in deciphering to what extent the non-coding space of contemporary genomes is “junk”^80,81^ or “polite”^82^.

## Methods

### Data sets

To study TADs and chromatin loops in human, coordinates from genome-wide chromatin interaction frequencies (Hi-C experiments) performed on human cell lines HMEC, HUVEC, IMR90, K562 and NHEK, were taken from Rao *et al*.^10^. Human and mouse genomic coordinates of Topologically Associated Domains (TADs) were taken from Dixon *et al*.^6^ and Pope *et al*.^83^, using comparative modENCODE/ENCODE (Encyclopedia of DNA Elements). Human and mouse isochores boundaries were adopted from Costantini *et al*.^84^. GC% variation was visualized using a colour map representing increasing GC% in the order (L1, L2, H1, H2, and H3 isochore families), deep blue (33–37%GC), light blue (37–41%GC), yellow (41–46%GC), orange (46–53%GC) and red (53–59%GC). These boundaries are applied to define L1-TADs, L2-TADs, H1-TADs, H2-TADs and H3-TADs. The human cell line data was converted to hg19 coordinates using UCSC liftOver when necessary.

Interchromosomal interactions data was from Quinodoz *et al*.^20^, the authors assigned genomic DNA to inactive/nucleolar or active/speckle hubs in mouse ES cells and human GM12878 cells at 1 Mb resolution. We also used a different set of interacting genomic intervals obtained by tethered chromosome conformation capture^27^, another method allowing the exploration of interchromosomal interactions. These authors calculated the Interchromosomal Contact Probability index (ICP), which is defined as the sum of interchromosomal contact frequencies divided by the sum of its inter- and intra-chromosomal contact frequencies. Therefore, ICP describes the propensity of a region to form interchromosomal contacts. This data is from GM12878 human lymphoblastoid cells.

### Clustering and identification of classes

To estimate the intra-loop patterns of GC variation, we divided the loops into two equal halves to quantify GC% increment/decrement (Fig. S1). The first half includes bin 1 to 50 and the second half includes bin 51 to 100. Thus, GC gradient of each half was identified by measuring the slope of the correlation coefficient (r) between the bin GC% and relative distance in each half of the TAD or loop. For each cell type in this study, a GC matrix of dimension Nx100 was thus obtained where N indicates the number of TADs identified in the particular cell type as rows, the 100 columns indicate the TAD/loop bins. Positive, negative or close to 0 values of the slope, respectively reflect increasing, decreasing or uncorrelated GC% *vs*. TAD/loop normalized coordinates. Ignoring orientation (equivalence of 5′ or 3′ gradient patterns), we defined six possible classes of loops or TADs; A (increasing or decreasing GC%), B (bell shape or peak), C (valley shape), B^−^ (half bell), C^−^ (half valley) and D (uncorrelated). Because GC-poor (L1, L2), and GC-rich (H1, H2, H3) isochore families generally define TADs^14^, the two properties (base composition and folding) are combined: GC-poor TADs (L1-TADs and L2 TADs) and GC-rich-TADs (H1-TADs, H2-TADs, H3-TADs). B (Bell shape) and C (valley shape) naming refers to compositional gradients within TADs.

Performing an unsupervised classification on the binned GC% variations across TADS/loops allowed us to verify if the above defined classes can be grouped in separate clusters. For this, we applied Principal Component Analysis (PCA) on the GC matrix for all cell types in study, using the R package FactoMineR^85,86^. The PCA clusters were identified using the R package factoextra^86^. Factors (F1, F2 and F3) explaining the majority of the variance will be used for visualization of TADs/loops clusters.

### Distribution of functional elements across TADs

To study functional aspects of TADs with respect to intra-TAD GC variation, genomic coordinates of protein coding genes were obtained from GENCODE^87^, and their expression levels in 27 tissues were collected from the GTEx portal; transcriptional activity is expressed as Transcripts Per Million (TPM) which is a normalization method for RNA-seq, it is read as “for every 1,000,000 RNA molecules in the RNA-seq sample, “*n”* came from this gene/transcript.”. Genomic coordinates of human super-enhancers were obtained from the database of super-enhancers in mouse and humans dbSUPER^88^, and of CTCF binding sites from CTCFBSDB 2.0^89^.

We next quantified the overlap between genomic coordinates of genes and loops boundaries using *bedtools*^90^. Only those overlaps were considered when the gene coordinates did not extend beyond the borders of the TADs. An index scale from 0.0 to 1.0 was used to assign relative positions of genes with respect to the TAD unit length; values in the extreme ends of this scale, i.e. 0.0–0.2 and 0.8–1.0 mean that the gene is located close to the borders of the TAD. Values in the middle of this scale, i.e. 0.3–0.7 mean that the gene is located around the centre of the TAD. The same approach was followed to analyse the distribution of super-enhancers and CTCF binding sites across TADs.

Distributions of housekeeping and tissue-specific genes within TAD classes were identified using the tissue specificity index (Tau)^91^. Genes with Tau value less than 0.3 were considered housekeeping genes, those with Tau value greater than 0.8 were considered tissue-specific.

## Supplementary information

Supporting information
