# Supplementary material for: DNA sequence-dependent chromatin architecture and nuclear hubs formation
Source: Sci Rep. 2019 Oct 10;9:14646. doi: 10.1038/s41598-019-51036-9 (PMC6787200; doi:10.1038/s41598-019-51036-9)
Supplement: Supplementary file 1 — Supporting information [file 41598_2019_51036_MOESM1_ESM.pdf]

DNA sequence-dependent chromatin architecture and nuclear hubs formation

Kamel Jabbari*, Maharshi Chakraborty and Thomas Wiehe.

Supporting information

This PDF file includes:

Six supplementary figures

Two supplementary tables

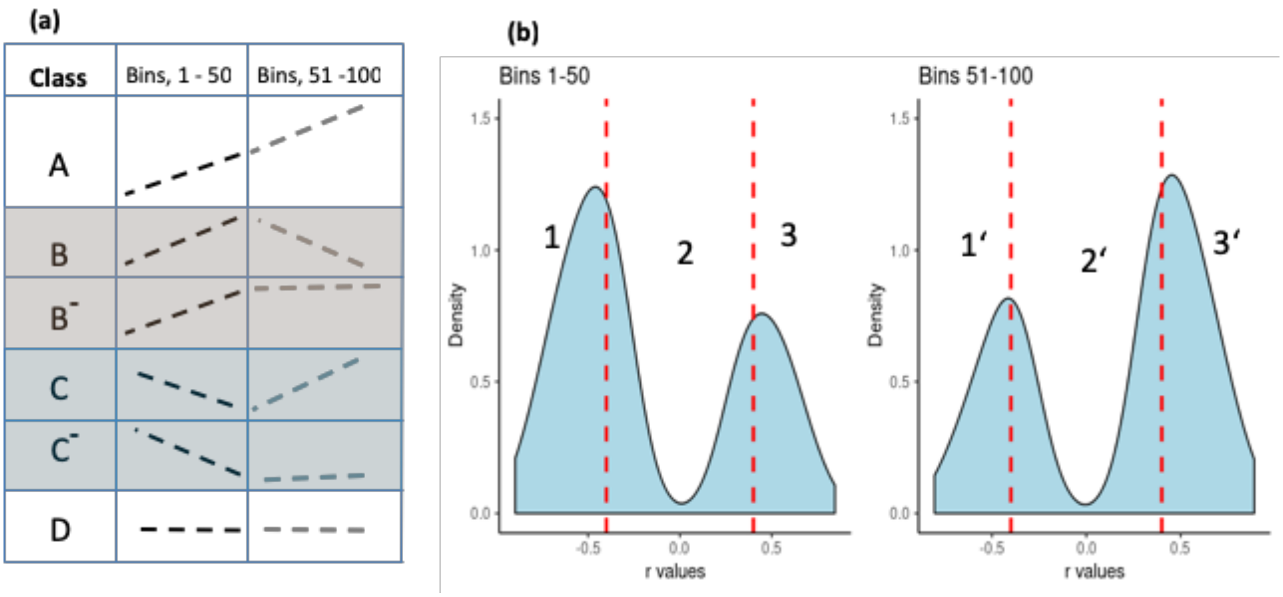

Fig. S1. (a) Schematic representation of the slope sign in left 50 bins and right 50 bins across TADs. Only one orientation is shown for A, B$^-$ and C$^-$. Same colour indicates similarity in GC profile between C and C$^-$ on one hand and B and B$^-$ on the other. **(b):** Distribution of r values with p < 0.05 of TADs from Dixon et. al. 1 and 1' indicate strong decreasing GC gradient (less than -0.4), 3 and 3' indicate strong increasing GC gradient (greater than 0.4), and 2 and 2' indicate a relatively flat GC profile (in between -0.4 and 0.4). Red lines indicate r value 0.4.

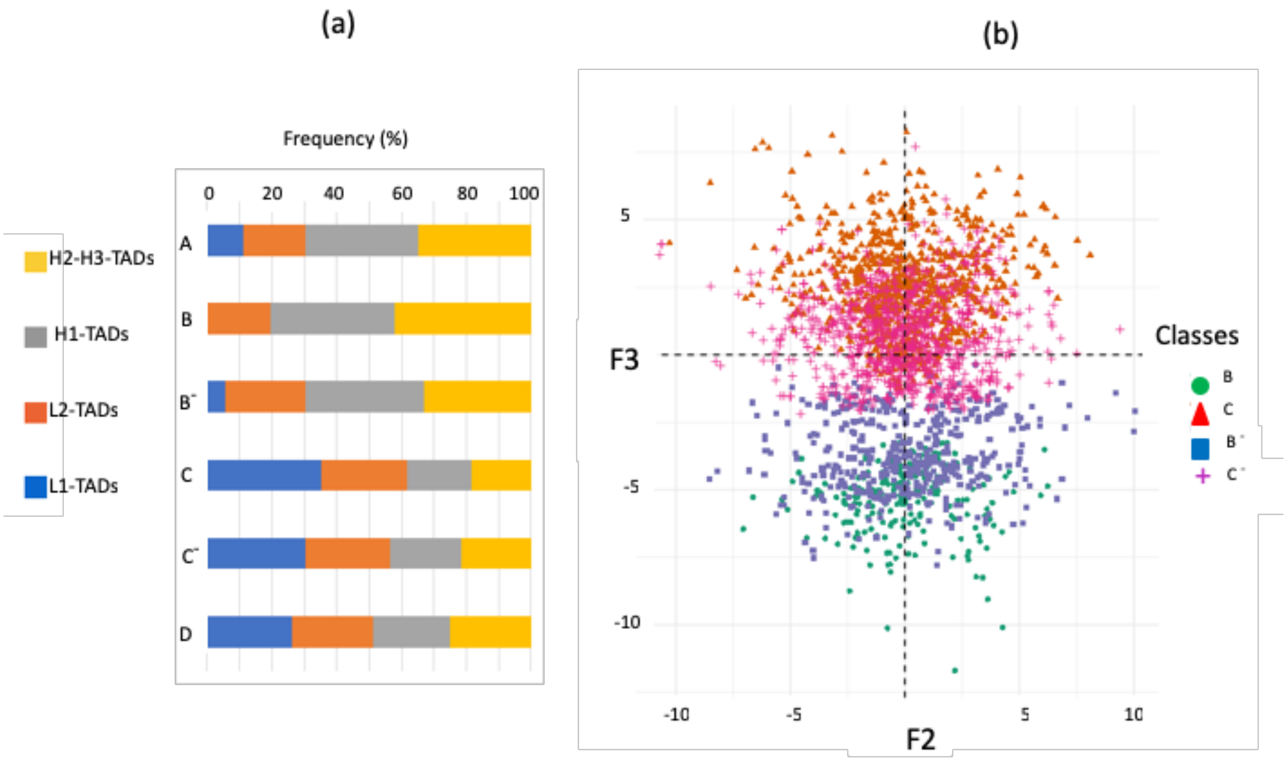

Fig. S2. (**a**) Distribution of all classes across TADs isochore families (L1 to H3). Classes B+B- and C+C- are most frequent in H1+H2+H3 TADs and L1+L2 TADs, respectively. (**b**) The distribution of classes B, B-, C and C- in the PCA (F2, F3) plane. The correlation between the average GC% of each TAD and F1 values was 0.99. B together with B- and C together with C- TADs are distinctly clustered, indicating that F2 and F3 explain substantial variation of GC% within each TAD (intra-GC variation).

	Class A	Class B	Class C	Class B-	Class C-	Class D
HMEC (Rao)	5.7	5.2	21.7	14.9	30.0	22.3
HUVEC (Rao)	3.3	7.4	31.3	13.5	26.1	18.1
NHEK (Rao)	3.6	6.5	21.2	15.3	28.9	24.1
IMR90 (Rao)	4.7	7.1	24.6	14.5	29.3	19.5
K562 (Rao)	3.8	6.0	29.7	13.5	28.3	18.5
TAD-IMR90 (Dixon)	4.2	7.4	27.9	18.0	22.9	18.0
TAD (Pope)	4.6	11.3	21.6	18.3	24.3	19.6
TAD-mES (Dixon)	5.5	6.4	30.0	13.8	26.8	17.1

Table S1: Quantities in percentage of TADs (Dixon and Pope data) and loops (Rao Data) in each class, from different data sources in our study (HMEC, HUVEC, IMR90, K562 and NHEK). These quantities were obtained after selecting Pearson "r" values as explained in Table S1.

To further investigate the classes, we wanted to check in which directions do the classes move amongst each other when the size of the loop is either increased or decreased. We first increased each loop in our study by 50kb on both 3' and 5' ends, and similarly decreased them by 50kb, and performed the same analysis as we did before with the raw data. As each loop now is 100kb larger or smaller, we increased the number of bins from 100 to 114 for the

enlarged loops and decreased them to 86 for the shortened ones.

**Class Change**	**A**	**B**	**C**	**B-**	**C-**	**D**
**A**	21.7	1.1	2.1	23.9	19.5	31.5
**B**	3.2	27.4	0.5	27.4	11.5	29.6
**C**	0.9	1.7	47.9	6.4	19.6	23.3
**B-**	3.7	9.5	3.9	42.3	8.9	31.6
**C-**	3.7	0.2	10.1	12.8	42.7	30.3
**D**	12.8	1.4	5.6	20.1	19.4	40,5

Table S2: indicates the average of percentage shift when the loops are increased or decreased in size. For every class, changing the size of the loop resulted in some transfer from a given class to the other. For class A we observed that the maximum shift was to class D (31.5%). Irrespective of extending or reducing the loops size, proportions of C, B^-, $C^-$and D classes are least affected, highlighting the fact that in terms of intra-GC variation they are the most stable. Expectedly, only ~ 1% inter-change between class B and class C is observed when the boundaries were changed, owing to their sharp intra-GC nature which are opposite to each other.

When the boundaries were shifted, a fraction of class B^- moved to class B with a ~2-fold bias compared to class C, and a substantial fraction of class C^- moved to class C with a ~10-fold bias compared to class B. This may be expected from the symmetric intra-GC spectrum of B^- and C^- describing half a peak or half a valley respectively. Furthermore, pairwise *t* test of average GC content of the TAD classes showed no difference between Class B^- and Class B (*p*-value = 1.0), whereas Class B^- and Class C are significantly different (*p*-value = 0.00023); likewise, Class C^- and Class B have divergent GC content (*p*-value = 0.00001) whereas Class C^- and Class C are similar (*p*-value = 1.0). The just-mentioned difference between TAD classes can also be seen when PCA is performed on classes B, C, B^- and C^-; B and B^- classes cluster along F3 PCA axis, opposite to C and C^- along the same axis.

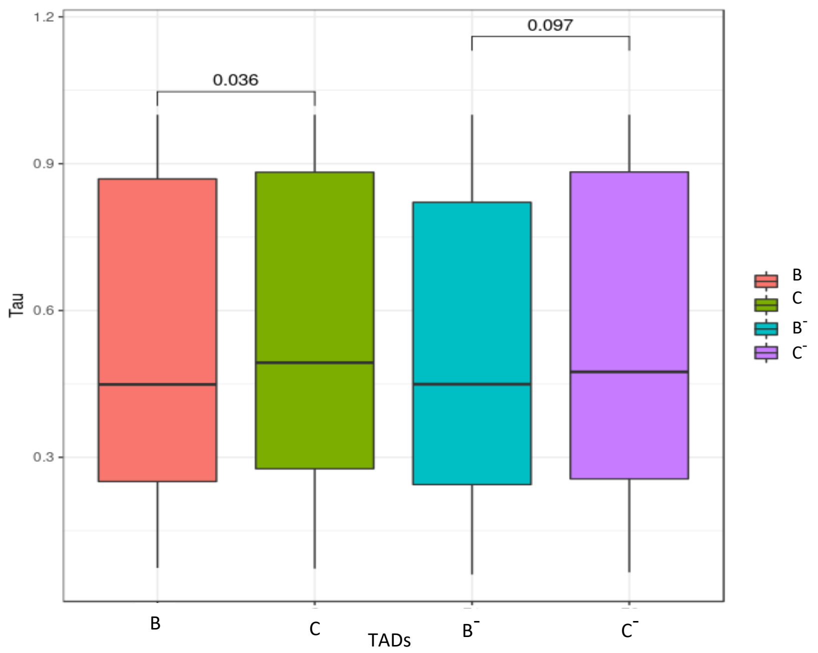

Fig. S3: Box plot showing that B-TADs harbour relatively more housekeeping genes than C-TADs (Wilcoxon rank test, *p*-value = 0.036); the same trend can be observed for class B⁻ compared to class C⁻, although with a Wilcoxon rank test *p*-value of 0.097.

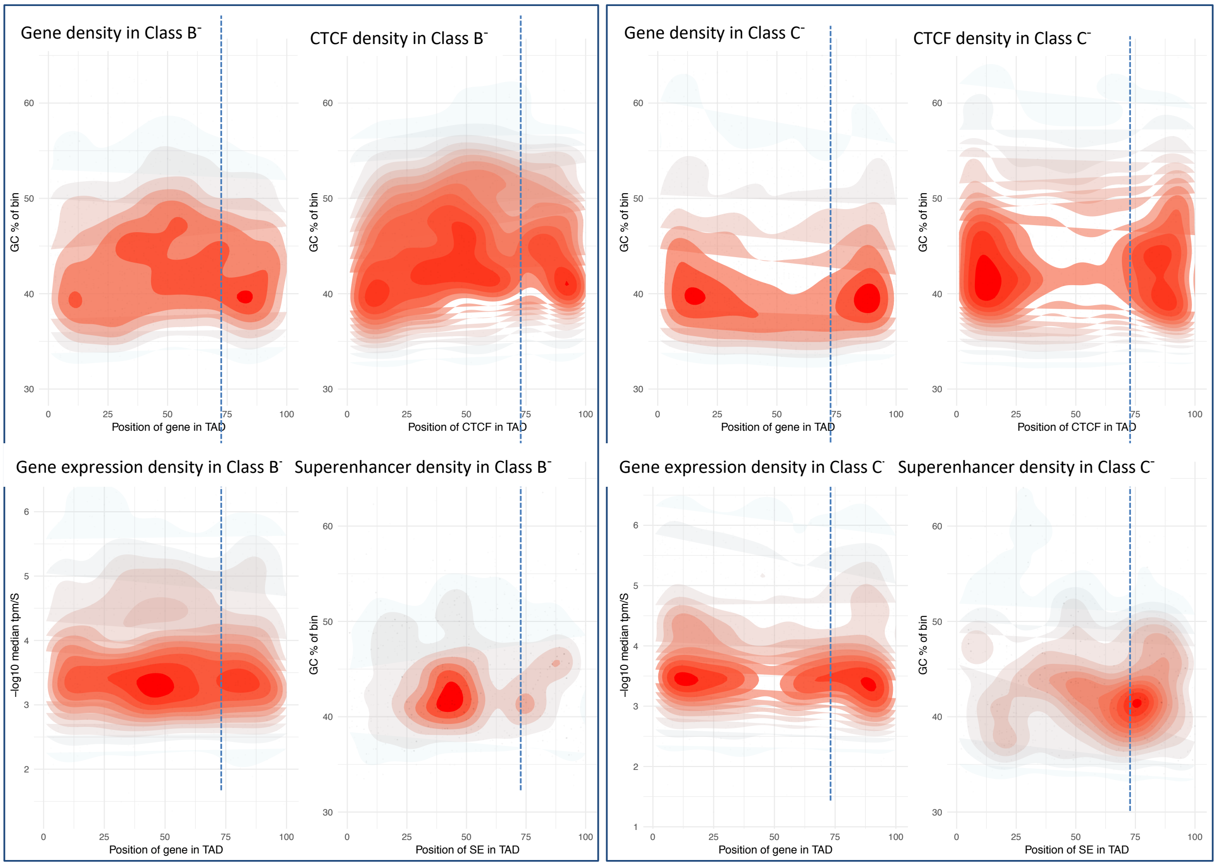

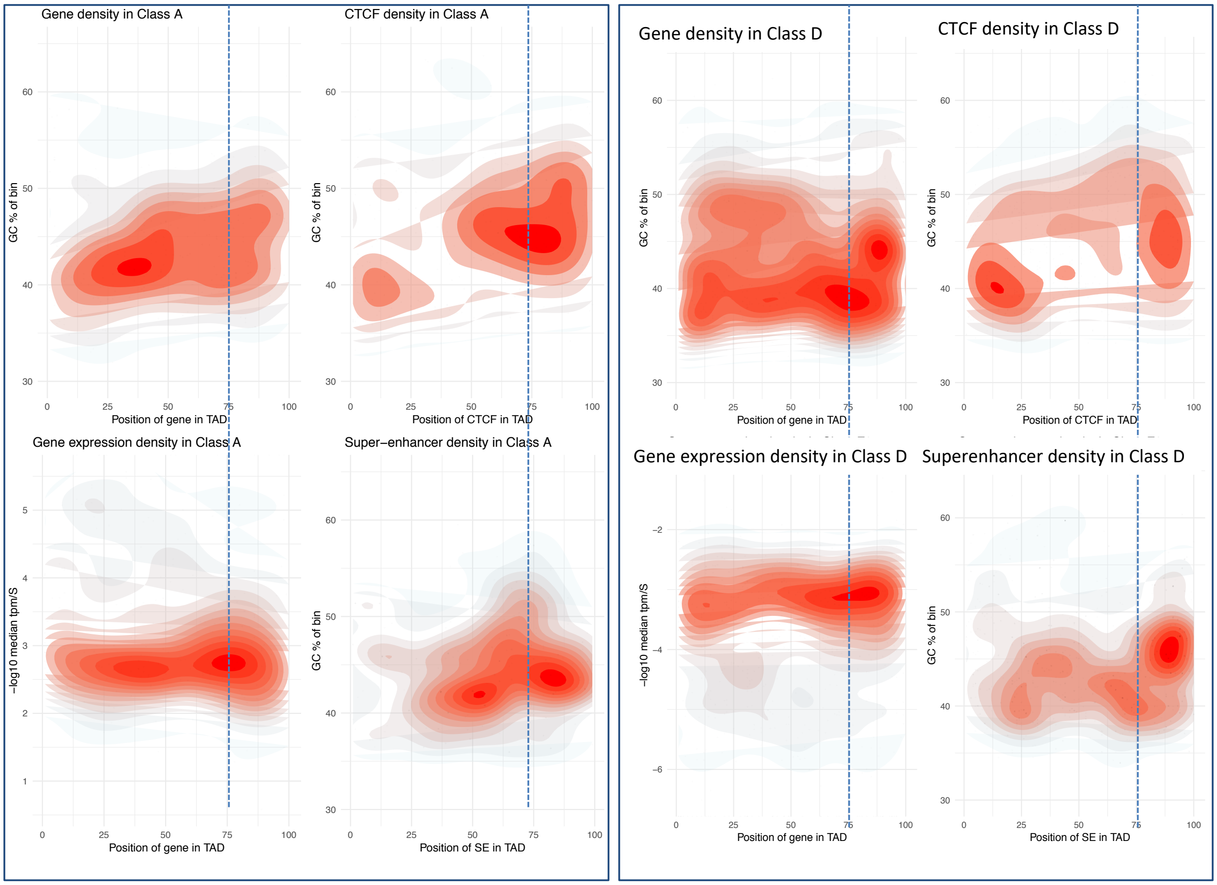

Fig. S4: Kernel density plots showing distribution of genes, $\log_{10}$ (mean TPM), super-enhancers and CTCF binding sites within class A, B$^-$, C$^-$ and D TADs. Red contours indicate high density of points whereas grey contours indicate lower density. Dotted lines mark the 75% bin, it points to the shift in density at the TADs border.

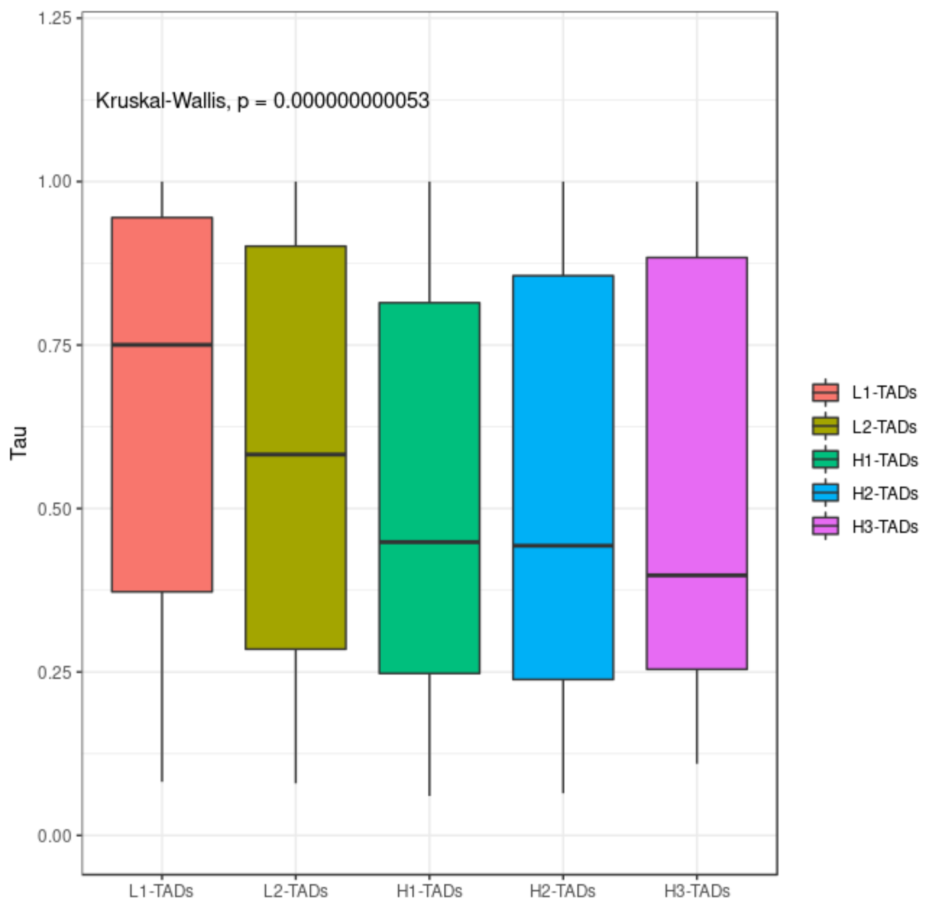

Fig. S5: Increase in housekeeping genes (low Tau value) from L1-TADs to H3-TADs. GC poor L1 and L2-TADs harbor genes with higher Tau value, *i.e*. tissue-specific genes. GC rich TADs (H1, H2 and H3) harbor genes with lower Tau values.

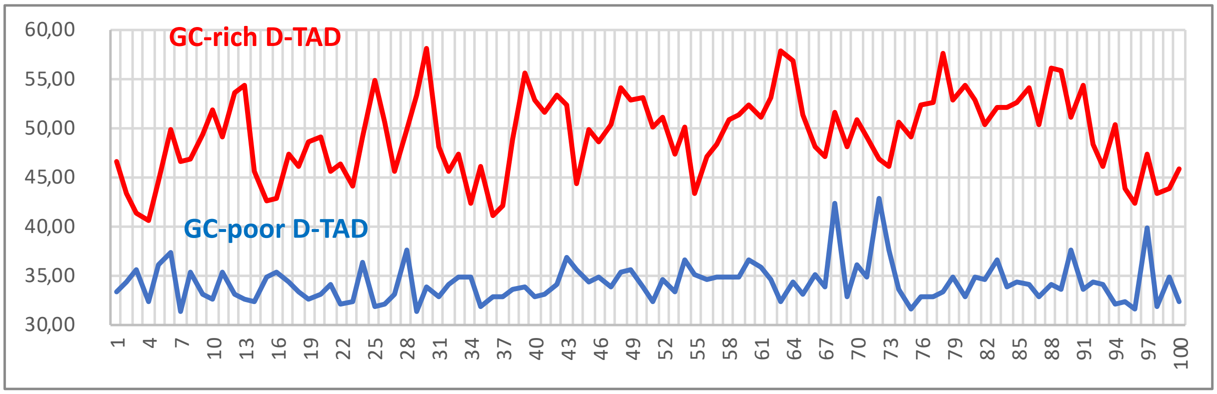

Fig. S6: GC profile of typical class D-TADs from L1/L2 and H2/H3 isochores. The higher heterogeneity of GC-rich TADs allows for multiple increased local flexibility.
